# Ethical challenges for the design and conduct of mega-biobanking from Great East Japan Earthquake victims

**DOI:** 10.1186/1472-6939-15-55

**Published:** 2014-07-04

**Authors:** Kenji Matsui, Shimon Tashiro

**Affiliations:** 1 Office for Research Ethics & Bioethics, the National Cerebral and Cardiovascular Center, Fujishiro-dai 5-7-1, Suita, Osaka 565-8565, Japan; 2 Office of Personal Information Protection, the National Cerebral and Cardiovascular Center Biobank, Fujishiro-dai 5-7-1, Suita, Osaka 565-8565, Japan; 3 Office for Promoting Medical Research, Showa University, Hatano-dai 1-6-8, Shinagawa-ku, Tokyo 142-8555, Japan

**Keywords:** Disaster, Research ethics, Biobank, Exploitation, Justice, Declaration of Helsinki

## Abstract

**Background:**

Amid continuing social unrest from the Great East Japan Earthquake and subsequent Fukushima nuclear accident of 2011, the Japanese government announced plans for a major biobanking project in the disaster-stricken areas, to be administered by the ‘Tohoku Medical Megabank Organization’ (ToMMo). This project differs from previous biobanking projects in that it 1) was initiated mainly to boost post-disaster recovery and reconstruction; and 2) targets the area’s survivors as its primary subjects. Here, we review the ethics of the ToMMo biobanking project within the wider context of disaster remediation.

**Discussion:**

Private citizens in the Tohoku region have criticized the project proposal, asking for further review of the ethics of targeting disaster survivors for this study. They claim the project violates the Declaration of Helsinki’s ethical provisions in that (1) government and university researchers initiated it without consulting any Tohoku survivors; (2) survivors already suffering extreme losses may view study involvement as meaningless or even undesirable, yet feel forced to participate in exchange for tenuous promises of future assistance, thus exploiting those most in need.

Although the ToMMo has promised certain future social benefits for the target population in exchange for participating in its biobanking research, it is questionable whether such research can address the immediate health needs of the Tohoku disaster survivors in any significant fashion. The ethics of recruiting still-struggling survivors is also questionable.

**Summary:**

This case analysis demonstrates that conducting a post-disaster biobanking project on survivors poses issues concerning potential exploitation and the just distribution of benefits and burdens. Though the ToMMo emphasizes the project’s importance for individual survivors and regional recovery, it is questionable whether such research can justly respond to the survivors’ immediate health needs and whether truly voluntary participation can be ensured in such a crisis. Our society must enhance this nationwide debate and reexamine our priorities for recovery in the disaster-stricken regions. We should evaluate both whether and how this project can truly contribute to the survivors’ quality of life.

## Background

On May 16, 2011 the Ministry of Education, Culture, Sports, Science and Technology (MEXT) and the Ministry of Health, Welfare, and Labours (MHLW) of Japan sent a joint administrative notice *“About Surveys and Research in the Disaster-stricken Regions”* to all domestic research institutions, universities, and related academic associations [1].

The notice requested that all surveys and other research conducted in the Great East Japan Earthquake-stricken areas:

1. Undergo advance review with a research ethics review committee, based on relevant governmental ethics guidelines;

2. Undergo advance consultation by relevant local governments within and surrounding disaster-stricken areas, with due regard for the needs of disaster survivors, while providing appropriate systematic healthcare and other services as needed; and

3. Avoid any overlap with similar existing surveys or research, so as to minimize their number.

This notice was prompted by issues raised by various academic associations, including the Japanese Society of Psychiatry and Neurology (JSPN). In an urgent official statement issued April 28, 2011, the JSPN claimed that many unnecessary, overly burdensome, ethically questionable, and/or exploitative research projects – not only medical but also social and behavioral – had been conducted in the stricken areas immediately after the disaster [2].

Among them, the largest and most controversial was the biobanking project, involving a genetic epidemiological study implemented by the Tohoku Medical Megabank Organization (ToMMo) at Tohoku University, one of Japan’s elite universities [3].

The most critical opposition to the ToMMo’s biobank project (ToMMo-BbP) came from a citizens’ group called the Miyagi Residents Center of the Post-Great East Japan Earthquake Recovery and Reconstruction Supports (MRC). On February 16, 2012, the MRC sent a letter of concern to all medical and healthcare institutions, political parties, and local governments in the Miyagi Prefecture entitled, “We require thoughtful re-consideration among concerned parties regarding the grand design of the ToMMo-BbP [4].” Their opposition stemmed mainly from what they perceived as significant conflicts between governmental aims, the ToMMo-BbP’s research goals, and the survivors’ immediate and long-term needs. That is, the government’s primary aim is reconstruction of quake-hit areas and economic recovery; ToMMo-BbP goals are to collect genetic samples and health data on survivors for a large-scale, decades-long, population-based genetic cohort study, including pediatric sub-cohorts, while the survivors’ priorities are to re-establish livelihoods and quality of life.

As background, population-based cohort studies usually target generally healthy communities and are conducted in areas where population flux is low. For instance, Iwate Prefecture (a disaster-stricken area) has a well-known, on-going cardiovascular cohort study, the Ohasama Study, which has monitored at-home blood pressures for about 30 years [5]. Among such cohort studies, those that include genetic data as potentially explanatory variables are called “genetic cohort studies.” There are at least 30 such genetic cohort studies in Japan, ranging from thousands to tens of thousands of cohorts [6]. Yet, quite atypically, the ToMMo-BbP was initiated by the Japanese government in order to boost post-disaster recovery and reconstruction, while targeting disaster survivors as its primary participants.

What are the consequences of targeting survivors rather than a healthy, unaffected population? In this paper, we examine this issue by reviewing the disaster’s aftermath with particular focus on the disaster regions’ in situ healthcare system and the details of the ToMMo-BbP study. We then discuss ethical issues with respect to the ToMMo-BbP, and conclude that the project’s design and implementation remain ethically problematic.

Note that this study refers only to the 2008 version of the Declaration of Helsinki (DoH), which was current when the ToMMo-BbP was initiated [7]. However, though the relevant sections (Provisions 17 and 26) of the DoH were indeed revised in 2013, the revisions remain altogether in accord with our present discussion. Also note that the forgoing analysis is based solely on documents and data sources currently published and publicly available. Thus, any aspects and/or changes to the ToMMo-BbP design, not available at the time of writing, are not reflected in the present manuscript.

## Discussion

### Case presentation: Great East Japan Earthquake and the ToMMo biobank project

More than 15,800 people died due to the earthquake on March 11, 2011. Over 2,600 people remain missing [8], and as of July 30, 2013 more than 290,000 remain displaced [9].

According to the MHLW’s report of July 11, 2011 [10,11], of the 380 hospitals in Tohoku’s three most-damaged prefectures – Iwate, Miyagi, and Fukushima – 10 (2.6%) were completely destroyed and 290 (76.3%) were severely damaged. Moreover, 83 (2.1%) of the 4,036 medical clinics and 83 (3.2%) of 2,597 dental clinics suffered complete collapse; 1,173 (29.1%) and 820 (31.6%) were partly destroyed; and 875 (12.1%) of the 7,206 social welfare facilities for children, elderly, and disabled persons were damaged or destroyed. About 30–40% of the damaged or destroyed facilities were, and many still are, closed or unable to receive patients. Furthermore, about 40% of the survivors, as well as 10% to 20% of local physicians report on-going health issues stemming from the disaster [12-14].

Long before the 2011 quake, along with rapid aging and depopulation Tohoku was already suffering from serious shortages in medical resources and personnel. Among Japan’s 47 prefectures, the number of physicians per 100,000 population, Iwate, Miyagi, and Fukushima ranked 37^th^, 27^th^, and 39^th^, respectively. Similarly, by nurses per 100,000, they ranked 14^th^, 33^rd^, and 38^th^, respectively [15].

Immediately after the disaster, the number of medical professionals dispatched as acute aid from intact medical institutions from all over Japan totaled about 10,000. However, the average daily number of active personnel was 500. And this number decreased significantly within two months of the disaster [11]. On the other hand, more than 1,200 local medical professionals in the stricken areas left their jobs [16]. Consequently, healthcare in already disadvantaged Tohoku has worsened substantially [14], especially in tsunami-ravaged coastal, rural regions remote from Tohoku’s central urban areas, such as Sendai City where Tohoku University is located.

In June 2011, during continuing social ferment stemming from the tsunami and ensuing Fukushima nuclear melt-down, the biobanking project was initially proposed and discussed by the Reconstruction Design Council and the Cabinet level Conference on Medical Innovation. The ToMMo was then established in February 2012 at Tohoku University and funded by the MEXT with 15.8 billion Yen (US$ 207 million) (which is 5.78% of the MEXT’s Science and Technology Budget in FY2011). Major funding was allocated through the government’s Special Reconstruction Budget, intended primarily to boost the economy and replace destroyed or damaged infrastructure.

The MEXT has stated that the primary goal of the ToMMo-BbP is to aid Tohoku’s economic recovery by establishing a center for a new healthcare industry, and promote nation-wide medical innovations, while creating a new, model healthcare system [17]. To achieve these goals, the ToMMo-BbP includes three major projects [18]: (i) creation of a massive genetic biobank with samples from 150,000 people, including disaster sufferers, healthy people, and children, with the aim of propelling nation-wide medical innovation, as well as establishing individualized medicine and preventive care through combined analyses of genetic and healthcare information; (ii) establishment of a model, regional healthcare IT network that shares patient healthcare records among medical institutions electronically; and (iii) the development of a pool of biomedical science professionals, such as clinical research coordinators, data managers, genetic counselors, bioinformation specialists, science communicators, and medical system engineers, needed to mount a nation-wide program of medical innovation.

To support the above projects, the ToMMo-BbP will collect biomaterials, including genetic samples and clinical information through an IT-networked, electronic patient healthcare records system in the damaged regions, from 80,000 disaster victims and 70,000 healthy individuals, derived from about 10,000 three-generation families (i.e. grandparents, parents, and grand-children, including expected newborns).

Prior to the 2011 earthquake, Tohoku was a region with low migration rates. There were many three-generation families in the same household or within close proximity. Therefore, establishing a large, multi-generational biobank and tracking its participants is easier in Tohoku than other areas of Japan. As incentives *in exchange* for biobank data, personal health data will be archived and free health-checkups will be offered. Also, young physician-researchers will be dispatched on one-year clinical fellowships to medical facilities in the disaster-stricken areas. Such fellowships will provide clinical services in three shifts of four months each in the stricken areas, help recruit biobank participants, then return to the University for 8 months to perform lab analyses and undergo further training in innovative clinical practice and skills. The first team of four physician-researchers was dispatched on October 2012 [19].

Moreover, new training courses will start at the Tohoku University Graduate School of Medicine and the newly-planned Graduate School of Public Health, to develop medical science professionals who are expected to embark on careers in medical research [18].

The ToMMo states that all of these biobank-mediated reconstruction programs will help restore the regional healthcare system, as well as boost Tohoku’s general economy.

### Grass-roots criticism and the Declaration of Helsinki

However, some argue that the project is fishing unethically in troubled waters – conducting a major biobanking study on survivors already laboring under the dual load of earthquake recovery and Tohoku’s pre-existing, substandard healthcare system. Taken together, this suggests that such cohorts are at greater risk of exploitation via such research. Dr. Hideki Komatsu, the Hospital Vice President of Kameda Medical Center in Chiba, which has provided substantial medical support to the disaster-stricken areas, has criticized the ToMMo-BbP and its priorities bitterly [20]. He views as inappropriate the use of funds from the Special Reconstruction Budget for this project because it will mainly benefit researchers and large businesses rather than its designated target population, the disaster survivors themselves. Such survivors, in real need of livelihood resuscitation, may never benefit in any significant fashion. Komatsu says, “Some may argue that survivors will also *prosper if the wind blows*. However, once we accept such logic, then any unrelated plan can be linked in turn to the Reconstruction projects.” Dr. Hidetoshi Mitobe, himself a disaster survivor and MRC organizer, also levied similar criticism. Mitobe claimed that [4,21]: (1) though the current focus should be on disaster recovery, the ToMMo-BbP was initiated by the national government and university researchers without consultation or dialog with either local governments around the disaster-stricken areas or the disaster survivors themselves; (2) whether the project is really desirable for survivors suffering great stress due to loss of homes, families, communities, and even hopes is quite dubious; (3) a project harboring such critical doubts regarding benefit to the target research population infringes on Provisions 17 and 26 of the Declaration of Helsinki (2008).

Provision 17 of the DoH (in the 2008 text) states that “Medical research involving a disadvantaged or vulnerable population or community is only justified if the research is responsive to the health needs and priorities of this population or community and if there is a reasonable likelihood that this population or community stands to benefit from the results of the research [7].” Indeed, Provision 17 strengthens Provisions 19 and 30 of the old DoH of 2000, and was developed in response to the 1990s controversy regarding randomized trials of a shorter-course regimen of zidovudine to prevent maternal-fetal Human Immunodeficiency Virus transmission in developing countries [22]. Two additional criteria: *responsiveness* and *reasonable availability*, were introduced to justify research on impoverished populations in developing countries. However, such criteria are not limited to developing nations. Although debate is on-going as to the interpretation and implications of these provisions [23-25], Mitobe claimed that Provision 17 renders the project in violation of the DoH and thus “unethical” [21].

Moreover, Mitobe claims that the project violates the Helsinki declaration because Provision 26 clearly states that, “When seeking informed consent for participation in a research study the physician should be particularly cautious if the potential subject is in a dependent relationship with the physician or may consent under duress. In such situations the informed consent should be sought by an appropriately qualified individual who is completely independent of this relationship.” Yet, the ToMMo-BbP plans to obtain consent in exchange for supplying the disaster-stricken regions with researcher-physicians whose primary aim is to conduct genetic research [21].

Meanwhile, prior to implementation, the MEXT formed an internal advisory committee to study the overall design of the ToMMo-BbP, including any ethical issues. The committee met five times between April and May 2012 and submitted final recommendations to the MEXT on June 7, 2012 [26]. Concerning ethical issues, they set forth four priorities: 1) assessing the project’s compliance with relevant ethics guidelines for research; 2) setting up an expert task force to formulate ethical policies for those involved in the project; 3) preparing a model for informed ‘broad consent’ for biobanking; and 4) ensuring that such informed consent is truly voluntary [26-30].

Their final recommendations emphasized the project’s need to consult with local communities and contribute to recovery in the quake-stricken areas. However, the main concern of Komatsu and Mitobe, potential violation of DoH provisions regarding exploitation of survivor subjects, was never discussed. In fact, *none* of the committee members referred to any of the relevant provisions of the DoH nor ethical issues concerning research on vulnerable populations. The committee simply emphasized a broad expectation that the project be a driving force for health care innovation and post-disaster recovery [26].

The ToMMo-BbP protocol was also reviewed and approved by an institutional ethics review committee at Tohoku University. Additionally the ToMMo itself set up a task force to exam ethical, legal, and social issues (ELSI) concerning their biobanking project. However, as of April 2014, the committee’s minutes, the ToMMo selection process, list of task force members, and task force discussions regarding the ELSI remain unavailable for public review [31].

### Ethical analysis I: validity of biobanking with disaster survivors – responsiveness and reasonable availability concerns

As stated in the 2008 DoH, provision 17, the *responsiveness* and *reasonable availability* require that research on disadvantaged populations prioritizes the subjects’ health needs and ensures that individual subjects retain reasonable access to research findings involving themselves [32-34]. Accordingly, some claim that the ToMMo-BbP will lessen the current inequities in Tohoku’s long-standing, substandard healthcare infrastructure, thereby achieving multiple local and national goals [17,35-40]. Supplemental to regular health checkups for disaster survivors, the project, while establishing a massive biobank for a genetic cohort study, will also prompt earlier detection and thus treatment of cancers, cerebro-cardiovascular diseases, diabetes, mental disorders and other common afflictions [41]. While monitoring each individual for decades, it will also collect and store biological materials and individual healthcare and lifestyle-related information, study correlations, and disseminate bulk research materials and information to various third parties. Through these activities the ToMMo-BbP project expects to establish a national foundation for future personalized and preventive health care.

With the reconstruction and creation of industrialized healthcare in Kobe after the Great Hanshin-Awaji Earthquake of 1995 as its model [42,43], the ToMMo-BbP also proposes to catalyze to be clustering of healthcare industries in Tohoku, as it gathers people, medical professionals, and capital investment, promotes local employment, and restores and improves health care in the disaster regions. Thus, the project may boost health care innovation and economic growth, not only in Tohoku but also nation-wide, as well as mitigate “brain drain” from the region by encouraging medical professionals and new graduates to remain in or relocate to Tohoku (Table 1). Accordingly, as Emanuel et al. suggests, in the broadest view, the ToMMo-BbP may justify itself by providing a “fair benefit” [23-25].

**Table 1:** Argued potential benefits from the Tohoku Medical Megabank Organization’s biobank project

***Beneficiary***	***Short-term benefits***	***Long-term benefits***
Individual research subjects	Free additional health examinations in addition to those given by the national health-checkup program	Individual health will be years-long watched over with loving eyes by the researchers
	Receiving individual results of the proven baseline health examinations	Discovered clinically-assessed individual genetic risk information as well as incidental findings will be returned
	Specialist referral services	
Local communities in the quake-hit regions	Deployment of young researcher-physicians for clinical services in three shifts of four months each in quake-hit areas	Repairment of the damaged local healthcare system
	Mitigation of brain drain	Solving a shortage of medical resources
	Promotion of local investment and employment	Improvement of the Tohoku’s long-standing substandard healthcare infrastructure
		Clustering healthcare industries
		Boosting the general local economy
		Attracting young people and medical professionals to move into the region
		Access to epidemiological findings on quake-/stress-induced diseases
National population	None	Economic recovery
		Promotion of medical innovations
		Creation of a model of a new healthcare system combined with the healthcare IT network
		Materialization of personalized medicine and prevention through a huge human genetic database and results of the cohort study

However this can be expected, provided such added benefits are reasonably foreseeable, and agreement on the amount and distribution of such benefits is achieved among all parties concerned. Certainly, the estimated impact on Tohoku’s economic growth and health care resources remains speculative. Indeed, 18 years after the disaster, the reality for Kobe is only partial success, with full recovery of the local economy and healthcare system still to be achieved [44].

Yet, uncertain execution and impacts cannot serve as an ethical hurdle to impede research. Rather, if there is good reason to believe that the balance between burdens and potential benefits seems sufficient, it may be considered as fair research [45]. In fact, the ToMMo-BbP’s anticipated benefit to Tohoku may be enormous, especially for long-term economic impact, and thus may justify the additional burden borne by Tohoku’s individual biobank contributors.

On the other hand, given the lack of primary focus, the project’s response to the immediate health-related needs and priorities of Tohoku’s most disadvantaged and thus vulnerable population, the disaster survivors, may never suffice (Table 2) [4,20,46]. Due to the project’s lengthy duration, the burden on individuals stemming from the collection and use of biodata is likely to be considerably higher than for shorter term projects. Moreover, as with nearly all types of biobank research, those who benefit first and foremost – in terms of health and economic considerations – will be our society as a whole, while direct benefits to individual project participants may be scarce. Such potential mismatch between burden-bearers and research beneficiaries may thus provide good reason to regard the project as ethically unjustified.

**Table 2:** Immediate health-related needs of the quake-survivors at the initial proposal of the ToMMo-BbP (June 2011)

Individual needs	Secured access to medical resources and healthcare services
	Specific treatments and care
	Real measures for individual livelihood rehabilitation
Communities’ needs	Rapid recovery of the collapsed local healthcare system
	Additional hundreds of battle-ready medical and healthcare staff
	More access to medical facilities and resources
	Recovery of livelihood including returning original homes/communities

*Abbreviation*: *ToMMo-BbP* the Tohoku Medical Megabank Organization’s biobank project.

That is, the basic conflict here is between attending to the immediate health care needs and concerns of disaster survivors, versus the need for long term economic and health care recovery in the disaster-stricken area and Japan as a whole. While recognizing the potentially positive, long term impact of economic development in the stricken area via investment in research infrastructure, it is also clear that several hospitals in the disaster-stricken areas have not recovered and many local clinics remain out of service. Thus, many survivors still have very limited access to medical resources. It is reported for instance that older people who have lost their families have become socially as well as medically isolated, and are therefore suffering unduly from declining health [12,47,48].

Thus, what is most needed is *immediate* disaster services, along with *achievable* concrete plans for securing access to sufficient health care and medical resources [4,49], rather than a *thin, speculative promise* of future health benefits [22]. Similarly, the immediate priority is rapid recovery for health care in the stricken regions, rather than the *eventual* improvement of Tohoku’s whole healthcare system.

Of course, one should acknowledge that the ToMMo-BbP has already begun supplying quake-struck areas with a few researcher-physician fellows on one-year rotations. There, they will provide medical guidance and collateral preventive heal care services. This will contribute to the health care recovery to some extent. Also, new masters and doctoral courses for nurses and other medical personnel will play a role in attracting young people to Tohoku.

Yet, disaster survivors need considerably more immediate help. Additional hundreds of battle-ready medical staff, more real physicians rather than researcher-physicians, more access to medical facilities and resources, and more specific treatment, rather than preventive medicine or health research [46,49-51]. Yet, the disaster survivors were never allowed to voice their feelings and opinions in determining the project’s grand design. Instead, university researchers determined the scope and framework of the project in its entirety before announcing it to a community still reeling from the loss and confusion caused by the disaster. As such, what the project has done and seeks to do seems clearly insufficient. Thus, it seems necessary to try to bridge the gap between the survivors’ health needs, project expectations, and the ToMMo’s ability to meet those more pressing needs [52]. Otherwise, the responsiveness and reasonable access concerns will remain unresolved. Only when more immediate needs are met will it be appropriate to consider a research project that may provide additional benefit to the stricken areas in terms of long-range infrastructure and development.

### Ethical analysis II: potential “benefits” and duress

Recently, as an ancillary benefit to contributors, several biobanks have begun to provide individual contributors with their research results – not only baseline healthcare data (e.g. blood pressure; urinary sodium) but also clinically-assessed genetic data [53,54]. Some biobanks have also promised to provide individuals with specialist referrals whenever significant medical conditions are encountered. Indeed, in addition to health-checkups, the ToMMo-BbP has actually promised to provide individual research results, along with specialist referrals for those with clinically significant medical conditions. The ToMMo even states that, through this project, they will *watch with caring eyes* the long-term health of disaster survivors [55,56]. Since direct benefits to project enrollees are otherwise scarce, offering such services seems wise, not only for ToMMo researchers seeking more contributors, but also desirable and workable as a strong incentive for potential contributors [57,58].

On the other hand, such promised ‘benefits’ will also strengthen the therapeutic misconception, or what Clayton and Ross term the “diagnostic misconception” [59]. Moreover, the promise of individual genetic results as a potential benefit disregards further consequences, such as that disclosing individual genetic data may elicit additional “anxiety and the burdens of follow-up [60].” And, in such damaged, limited healthcare environs disaster survivors need immediate access to healthcare. Thus, they may tend to view any healthcare as better than none. So, it becomes questionable whether those with an impulse to decline feel they can really afford to reject project participation. They may also feel obligated or be unduly pressured by the surrounding community, especially when overall benefits to the community require strong, individual, project participation. Certainly, such vulnerability by itself may not constitute unjust exploitation [25]. However, the potential remains for “imposing an unfair share of the burden of research participation” on a distressed population [61]. Also, given the level of shock, numbness and despair, due to their devastation, it is possible that such subjects “may *be* exploited without *feeling* particularly exploited, or without believing they are being exploited [62].”

To ameliorate such concerns the ToMMo prepared two distinct consent forms, one for health-checkups, the other for biobanking research. However, as administered by researchers intent on gathering such data, it remains dubious that such forms alone can function to avoid “participation under duress”. Rather, an alternative protocol that completely separates recruitment staff from biobanking researchers or health service providers should be considered, following due consideration and proactive consultation with potential subjects and other relevant parties.

### Current project status and further considerations

Despite many remaining ethical issues concerning its design, the recruitment phase of the ToMMo-BbP began on May 20, 2013. As of September 2013, more than 10,000 participants have been recruited with a consent rate of over 60% [63,64]. Because it is regarded partly as a social venue or infrastructure for post-disaster restoration and reconstruction, our society must engage in the national debate about ToMMo-BbP. We need to reexamine our priorities for both recovery in the disaster-stricken regions and restoring the livelihoods of suffering survivors, and decide in what way the project can truly contribute to this purpose.

Although the project was launched with little consideration for or consultation with disaster survivors, project researchers are now working via trial and error to establish the right path. They have created a public relations section within the organization to promote communication with local communities [65]. Recently, they have also begun to invite external research ethicists (including the authors) for frank and open dialogue regarding the project’s ethical issues [66]. Such efforts are appreciated, and one may expect, through sincere discussion and dialogue with survivors about the project’s remaining problems and its desired future, that the project will improve ethically and fulfill more of the real, immediate needs of the disaster survivors and their stricken communities.

Finally, this case analysis may facilitate further discourse regarding the responsiveness and reasonable availability requirements, originally developed for clinical trials in developing nations, via lengthy discussions within the international ethics research community. First, it shows clearly that these requirements can raise major research issues, not just in developing nations but also in the aftermath of natural disasters occurring in developed nations [62]. Second, it demonstrates practical and political difficulties in applying the requirements as applied to biobanking research, since such requirements were developed primarily within the context of clinical trials. Finally, this case also raises the question of whether knowledge acquired by research on the chronically disadvantaged can apply to the temporarily disadvantaged, i.e., disaster survivors whose prior life circumstances may be restored within a reasonably foreseeable future. This final point has not been well-studied, and thus needs careful, future analysis.

## Summary

Immediately after the Great East Japan Earthquake in 2011, the government initiated a large-scale biobanking project through Tohoku University to service the disaster-stricken areas. This project began without consulting the disaster survivors themselves. The goals were to both boost post-disaster recovery and to promote nation-wide medical innovation. Advisory committees were formed to examine the ELSI of the project at both government and university levels. However, our ethical analysis of this project demonstrates that it still has many unresolved ethical problems in its design and its implementation of the just distribution of benefits. It may also unduly burden and/or potentially exploit a vulnerable population in crisis. Although the government and university researchers emphasize the project’s anticipated role in societal recovery within the quake-damaged regions and its potential benefit for individual participants, it is highly questionable whether such decades-long biobanking research, with only a distant promise of speculative future social benefits, can be justified in lieu of a more timely response to the immediate health needs and priorities of the disaster survivors. It is also questionable whether duress concerns can be eliminated during the recruitment process conducted in the midst of such a crisis. Therefore, before the project proceeds further, we recommend that the ToMMo-BbP and our society as a whole open a national discussion regarding the project ethics, and that we reexamine what can and should be our immediate response and first priorities in support of the disaster survivors’ immediate needs.

## Abbreviations

MEXT: Ministry of Education, Culture, Sports, Science and Technology; MHLW: Ministry of Health, Welfare, and Labours; JSPN: Japanese Society of Psychiatry and Neurology; ToMMo: Tohoku Medical Megabank Organization; ToMMo-BbP: ToMMo’s biobank project; MRC: Miyagi Residents Center of the Post-Great East Japan Earthquake Recovery and Reconstruction Supports; DoH: Declaration of Helsinki.

## Competing interests

Kenji Matsui, and Shimon Tashiro declare no competing interests.

## Authors’ contributions

KM has contributed to the conception, design, intellectual analysis, acquisition of references, draft writing, and revision of the manuscript. ST has contributed to the intellectual analysis, and critical revision of the manuscript. Both have given final approval of the submitted version and are responsible for all aspects of the work.

## Pre-publication history

The pre-publication history for this paper can be accessed here:

http://www.biomedcentral.com/1472-6939/15/55/prepub
